# Ultra-Low Power High Temperature and Radiation Hard Complementary Metal-Oxide-Semiconductor (CMOS) Silicon-on-Insulator (SOI) Voltage Reference

**DOI:** 10.3390/s131217265

**Published:** 2013-12-13

**Authors:** El Hafed Boufouss, Laurent A. Francis, Valeriya Kilchytska, Pierre Gérard, Pascal Simon, Denis Flandre

**Affiliations:** ICTEAM Institute—Electrical Engineering, Université catholique de Louvain, Maxwell Building, Place du Levant 3, B-1348 Louvain-la-Neuve, Belgium; E-Mails: laurent.francis@uclouvain.be (L.A.F.); valeriya.kilchytska@uclouvain.be (V.K.); pierre.gerard@uclouvain.be (P.G.); pascal.simon@uclouvain.be (P.S.); denis.flandre@uclouvain.be (D.F.)

**Keywords:** biomedical, high temperature, CMOS subthreshold regime, total ionized dose, ultra-low power, voltage reference

## Abstract

This paper presents an ultra-low power CMOS voltage reference circuit which is robust under biomedical extreme conditions, such as high temperature and high total ionized dose (TID) radiation. To achieve such performances, the voltage reference is designed in a suitable 130 nm Silicon-on-Insulator (SOI) industrial technology and is optimized to work in the subthreshold regime of the transistors. The design simulations have been performed over the temperature range of −40–200 °C and for different process corners. Robustness to radiation was simulated using custom model parameters including TID effects, such as mobilities and threshold voltages degradation. The proposed circuit has been tested up to high total radiation dose, *i.e.*, 1 Mrad (Si) performed at three different temperatures (room temperature, 100 °C and 200 °C). The maximum drift of the reference voltage *V_REF_* depends on the considered temperature and on radiation dose; however, it remains lower than 10% of the mean value of 1.5 V. The typical power dissipation at 2.5 V supply voltage is about 20 *μ*W at room temperature and only 75 *μ*W at a high temperature of 200 °C. To understand the effects caused by the combination of high total ionizing dose and temperature on such voltage reference, the threshold voltages of the used SOI MOSFETs were extracted under different conditions. The evolution of *V_REF_* and power consumption with temperature and radiation dose can then be explained in terms of the different balance between fixed oxide charge and interface states build-up. The total occupied area including pad-ring is less than 0.09 mm^2^.

## 1. Introduction

**Figure 1.**
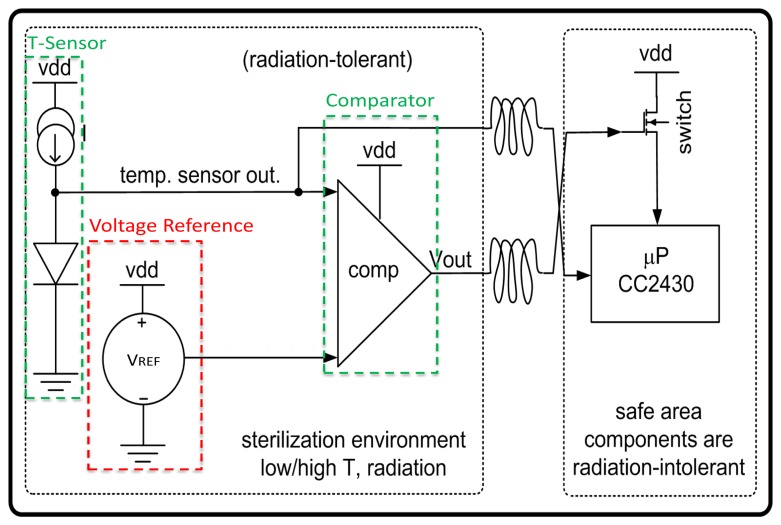
Biomedical microsystem adapted from [1].

Silicon circuits are increasingly sought by biomedical applications, such as radiation detectors, breathing sensors and temperature sensors. These applications often require ultra-low power circuits, sometimes also robust to harsh environments. Figure 1 shows our previous development [1] of an ultra-low power microsystem, composed of a temperature sensor, a comparator and a voltage reference. This microsystem aims at temperature sensing in the harsh conditions used in medical sterilization such as high total ionized dose radiation (TID) or high temperature. It has three main functions: detecting a user-defined temperature threshold *T*_0_, generating a wake-up signal that turns on a data-acquisition microprocessor above *T*_0_, and measuring temperatures above *T*_0_. The microsystem was developed using a suitable and robust technology, *i.e.*, 1 *μ*m partially-depleted (PD) Silicon-on-Insulator (SOI). Such technology is often used in harsh environments applications because of its attractive features; extended range of working temperature (up to 300 °C), reduced parasitic effects, low-power, high speed and lower sensitivity to transient radiation effects comparing to other technologies [2]. However, during measurements, the voltage reference circuit was the weak point of the microsystem and showed a large shift in the measured reference voltage with radiation of up to 900 mV at 1 Mrad (Si) [3]. To overcome this limitation, a new design of an ultra-low power and harsh-environment immune voltage reference is performed. We firstly choose to port the design to a more suitable SOI industrial technology with Complementary Metal-Oxide-Semiconductor (CMOS) process node of 130 nm featuring a much reduced gate and buried oxides thickness and hence TID degradation [4]. Secondly, we improved the architecture by introducing a cascode bias stage for enhanced stability, and a new start-up design to avoid start deficiency at low temperature and extreme corners (resulting in high *V_th_*). This proposed circuit is optimized to work in the subthreshold regime of the transistors in order to achieve ultra-low power dissipation (less than 100 *μ*W) at high temperature (up to 200 °C).

In this paper, we firstly present the design of the voltage reference circuit. Then, we detail the experimental results of the voltage reference under large range of temperature and under combined high temperature and radiation influence. Lastly, we explain the impact of the combination of high total ionizing dose and high temperature on the proposed circuit and draw conclusions.

## 2. Basic Concepts

### 2.1. Voltage Reference Circuit Description

**Figure 2.**
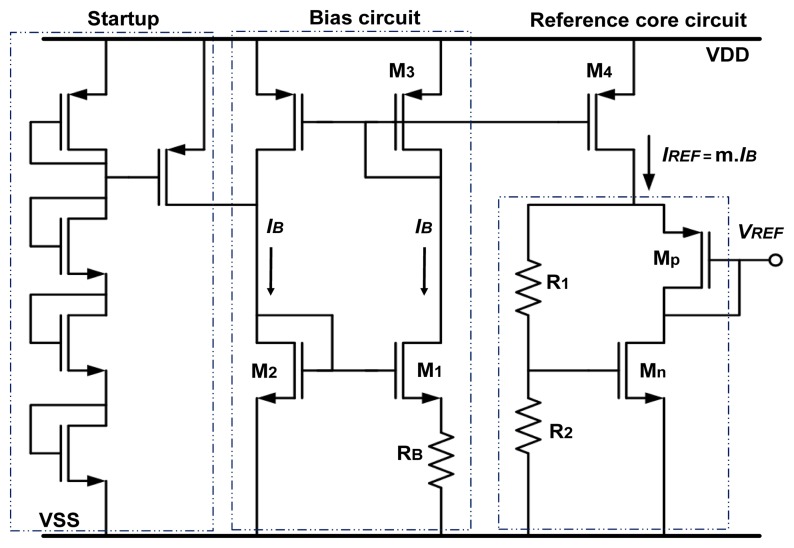
Voltage reference circuit adapted from [3].

The voltage reference is a CMOS circuit (Figure 2) based on the gate-source voltage difference between a pair of P-type MOS (PMOS) and N-type (NMOS) transistors (*M_p_* and *M_n_*) biased by a proportional to absolute temperature (PTAT) current *I_REF_* (Equation (8)). Differently to the initial work described in [5], our design was: (1) improved by introducing a cascode bias stage for enhanced stability and a new start-up design to avoid start deficiency at extreme temperature and process corners (resulting in high *V_th_*); (2) extended to operate in a large temperature range from −40 to 200 °C; (3) conceived to limit the power dissipation in harsh environments, using transistors optimized to work in subthreshold regime.

In the next paragraph, operation of our previous circuit [3] is reviewed, and in the next sections, an improved architecture is proposed.

### 2.2. Design Principles

From the analysis of the circuit described in Figure 2, the voltage reference value *V_REF_* and the bias current *I_B_* are obtained as:

(1) $$V_{\text{REF}}=\left(1+\frac{R_{1}}{R_{2}}\right)\cdot V_{\text{GSn}}-\left|V_{\text{GSp}}\right|$$

(2) $$I_{B}=\frac{V_{GS2}-V_{GS1}}{R_{B}}$$

where *V_GSn_, V_GSP_, V_GS_*_1_ and *V_GS_*_2_ are the gate-source voltages for transistors *M_n_, M_p_, M*_1_ and *M*_2_, respectively. For NMOS and PMOS transistors working in the subthreshold regime, the *I* – *V* characteristics can be expressed by [6]:

(3) $$I_{Dn}=\beta_{n}\cdot U_{T}^{2}\cdot \text{exp}\left(\frac{V_{GS}-V_{\text{thn}}}{n\cdot U_{T}}\right)\cdot \left[1-\text{exp}\left(\frac{V_{DS}}{U_{T}}\right)\right]$$

(4) $$I_{Dp}=\beta_{p}\cdot U_{T}^{2}\cdot \text{exp}\left(\frac{V_{SG}-\left|V_{\text{thp}}\right|}{n\cdot U_{T}}\right)\cdot \left[1-\text{exp}\left(\frac{V_{SD}}{U_{T}}\right)\right]$$

where *β_n_* = *μ_n_.C_ox_*.(*W_n_/L_n_*) and *β_p_* = *μ_p_.C_ox_*.(*W_p_/L_p_*), *μ_n,p_* is the electron and holes mobilities in the channel, *C_ox_* is the oxide capacitance per unit area, *V_thn_* and *V_thP_* are the threshold voltages of NMOS and PMOS respectively, *W_n_* (*W_p_*) and *L_n_* (*L_p_*) are the channel width and length for NMOS (PMOS), respectively. *U_T_* = *k_B_.T/q* is the thermal voltage (*k_B_* is the Boltzmann constant, *q* the elementary charge and *T* the absolute temperature), *n* is the subthreshold slope parameter, *V_GS_* and *V_DS_* (*V_SG_* and *V_SD_*) are the gate-to-source and drain-to-source voltages respectively for the NMOS (PMOS) transistor. For |*V_DS_*| > 4*U_T_*, the drain current *I_D_* becomes almost independent of the drain-to-source voltage [7], so that from Equations (3) and (4) we can extract:

(5) $$V_{\text{GSn}}=n\cdot U_{T}\cdot ln\left(\frac{I_{Dn}}{\beta_{n}\cdot U_{T}^{2}}\right)+V_{\text{thn}}$$

(6) $$\left|V_{\text{GSp}}\right|=n\cdot U_{T}\cdot ln\left(\frac{I_{Dp}}{\beta_{p}\cdot U_{T}^{2}}\right)+\left|V_{\text{thp}}\right|$$

In Figure 2, if *k* is the size ratio (*W*_1_.*L*_2_*/*(*L*_1_.*W*_2_)) of transistors *M*_1_ and *M*_2_, *m* the current copy ratio in the output stage (*I_REF_* = m.*I_B_*) and using Equations (2) and (5), the bias current *I_B_* is given by:

(7) $$I_{B}=n\cdot U_{T}\cdot \left(\frac{ln\left(k\right)}{R_{B}}\right)$$

(8) $$I_{\text{REF}}=m\cdot n\cdot U_{T}\cdot \left(\frac{ln\left(k\right)}{R_{B}}\right)$$

Assuming that the current through the resistances *R*_1_ and *R*_2_ is negligible and using Equations (1), (5) and (6) we obtain the following expression of *V_REF_*:

(9) $$V_{\text{REF}}=n\cdot U_{T}\cdot \left[\ln\;\left(\frac{\beta_{Mp}}{\beta_{Mn}}\right)+M_{r}\cdot \ln\;\left(\frac{n\cdot m\cdot \ln\left(k\right)}{R_{B}\cdot \beta_{Mn}\cdot U_{T}}\right)\right]+\left(1+M_{r}\right)V_{\text{thn}}-\left|V_{\text{thp}}\right|$$

with *β_Mn_*_(_*_p_*_)_ = *μ_n_*_(_*_p_*_)_.*C_ox_*.(*W/L*)*_n_*_(_*_p_*_)_ for the output transistors *M_n_* and *M_p_* respectively (Figure 2). These *β* parameters (∝*T*^–^*^α^* with α ≥ 1.5) together with the parameter *n* and the threshold voltage *V_thn_* and *V_thp_* have a temperature dependency [8,9]. *M_r_* is the resistors ratio (*R*_1_*/R*_2_).* R_B_* is a non-silicided polysilicon resistance, showing almost negligible temperature dependency in the simulations.

## 3. Circuit Realization

**Figure 3.**
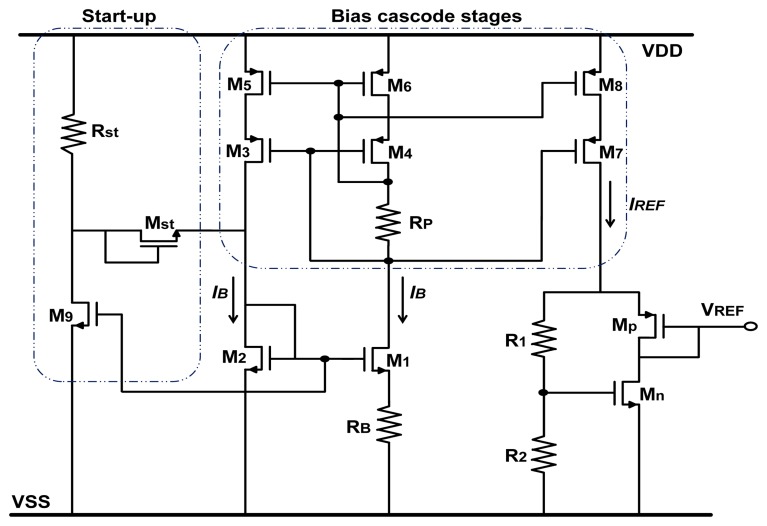
Proposed voltage reference circuit.

A new design of the voltage reference (Figure 3) was developed with a cascoded current source, to reduce the *V_REF_* variations due to the process corners, supply voltage variation and mainly the radiation effects caused by single ionized particles [10]. As it is based on the same principle as the previous design [3], the voltage reference expression *V_REF_* remains unchanged (Equation (9)).

### 3.1. Design Phases

#### 3.1.1. Technology Characterization at High Temperature

**Figure 4.**
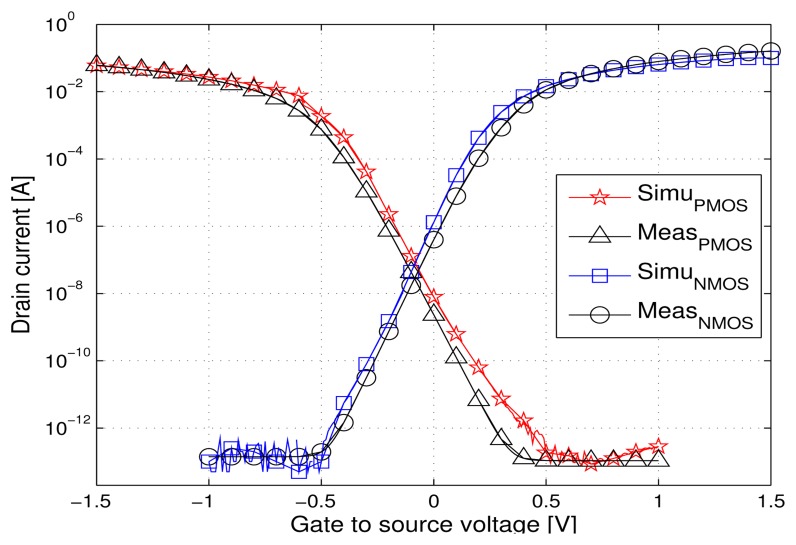
Measured and simulated *I_D_* versus *V_GS_* curves at 25 °C, *V_DS_* = 1.5 V, W = 1 *μ*m, L = 0.28*μ*m.

**Figure 5.**
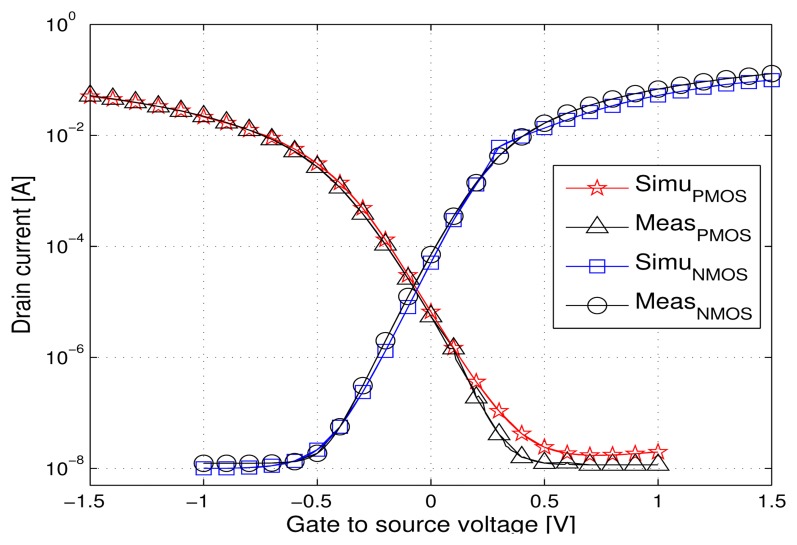
Measured and simulated *I_D_* versus *V_GS_* curves at 200 °C, *V_DS_* = 1.5 V, W = 1 *μ*m, L = 0.28*μ*m.

The voltage reference circuit was developed based on the analog MOSFETs of an industrial 130 nm PD SOI CMOS featuring a 5 nm gate oxide thickness and 280 nm minimum length. The SPICE (BSIM3SOI) models available for this technology are commercially validated up to 150 °C. We checked the models validity up to 200°C, by comparing the typical DC *I_D_*(*V_GS_*) characteristic of a MOSFET transistor extracted by simulations and from measurements for different temperatures (25, 150, 175 and 200 °C). Figures 4 and 5 show two examples of the good agreement between simulated and measured curves, for NMOS and PMOS transistors at 25 °C and 200 °C, in the subthreshold region of interest. The slight discrepancies for the PMOS transistor can be neglected since the circuit design has to cope with much larger variations due to process, temperature and radiations.

#### 3.1.2. Design Optimization

The design optimization consists in determining the bias current *I_B_* and the transistor and resistor sizes (in Figure 3) to minimize the *V_REF_* variation with the device physical parameters as given by the previous equations and process corners. The temperature dependence of the voltage reference must be as low as possible. The aforesaid dependence is expressed by evaluating the temperature coefficient (*TC*) defined by the following expression:

(10) $$\left[TC\right]=\frac{1}{V_{\text{REF}}}\times \left[\frac{dV_{\text{REF}}}{dT}\right]$$

In order to minimize the *TC* coefficient for our design, we have extracted temperature dependencies of the physical parameters, computed Equation (10) and searched the best design parameters (*W*_1_, *W*_2_, *L_n_, m, M_r_, W_n_, W_p_* and *R_B_*). However this first guess does not take into account the process corners. Next, extensive ELDO (Mentor Graphics) simulations were performed for the different process corners in a temperature range of −40 to 200 °C.

### 3.2. Design Robustness Against Radiation Effects

**Figure 6.**
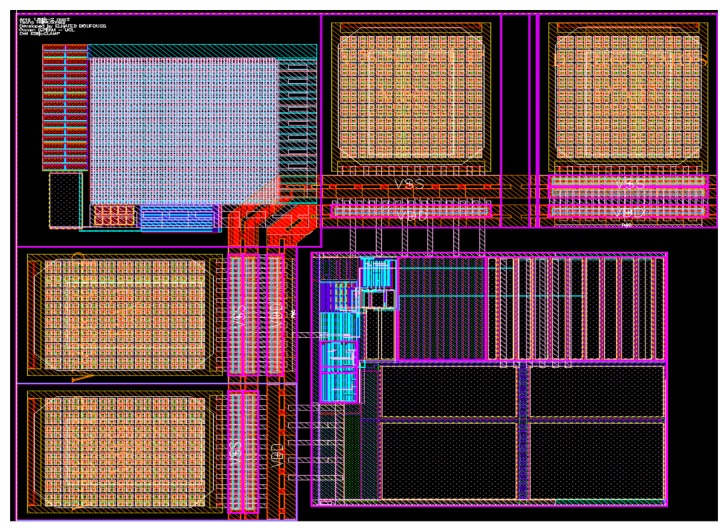
Final layout of the voltage reference.

To take into account the variations due to radiation effects, additional constraints were considered as follow:

- The designed circuit was checked with custom model parameters of transistors including TID effects on transistors [11] (up to 30% mobilities degradation and negative voltage threshold shifts of 100 mV).
- Using transistors with a body contact to limit the effect of the parasitic bipolar possibly created by radiation [10].
- Choosing relatively long transistor to assure lower leakage current and lower threshold voltages shift, which may appear as a result of TID and high-temperature effects [2,12,13].
- The circuit layout is further carried out by paying attention to critical components; thus all matched device pairs were realized by a centroid implementation, including the necessary dummies (Figure 6).

Table 1 gives the bias setting and different dimensions of transistors and resistors used in the proposed voltage reference circuit.

### 3.3. Simulations Results

Simulations are performed for five different process corners and a custom model parameters of transistors including TID effects, namely:

- Typ: typical corner (typical |*V_th_*| values)
- FFA: Fast PMOS and NMOS (min |*V_th_*| values) with minimum resistances values
- SSA: Slow PMOS and NMOS (max |*V_th_*| with maximum resistances values
- FSA and SFA: are the crossed cases (fast and slow)
- RAD: Custom model parameters of transistors including TID effects (30% degradation of the mobility and a threshold voltage shift of 100 mV).

**Figure 7.**
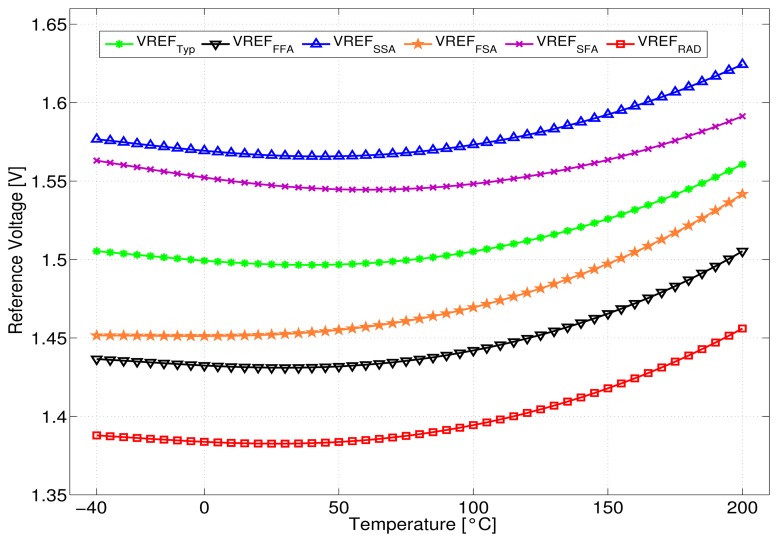
Simulated reference voltage *V_REF_* versus temperature and process corners.

As shown in Figure 7, the reference voltage *V_REF_* versus temperature range of −40 to 200 °C gives an average voltage of 1.5 V for Typical corner, with a variation of ±8% over all process corners and temperature/radiation conditions. More specifically, lower |*V_th_*| values lead to a lower *V_REF_* at a given temperature according to Equation (9), whereas the temperature dependence depends on the exact balance between the n, *U_T_* and the *V_th_* terms.

## 4. Experimental Results

The main purpose of our voltage reference is to be used in a harsh biomedical sterilization conditions. To validate this feature, first the voltage reference circuit was measured in a large range of temperatures from −40 to 200 °C. Subsequently, other chips have been tested during irradiation at different temperatures, using a small ceramic heater resistor placed under chips. Irradiation was performed at the Cyclotron facility of UCL with Gamma-rays, using a Cobalt source (^60^*Co*). The next sections present the measured results for a power supply of 2.5 V.

### 4.1. Temperature Measurements

**Figure 8.**
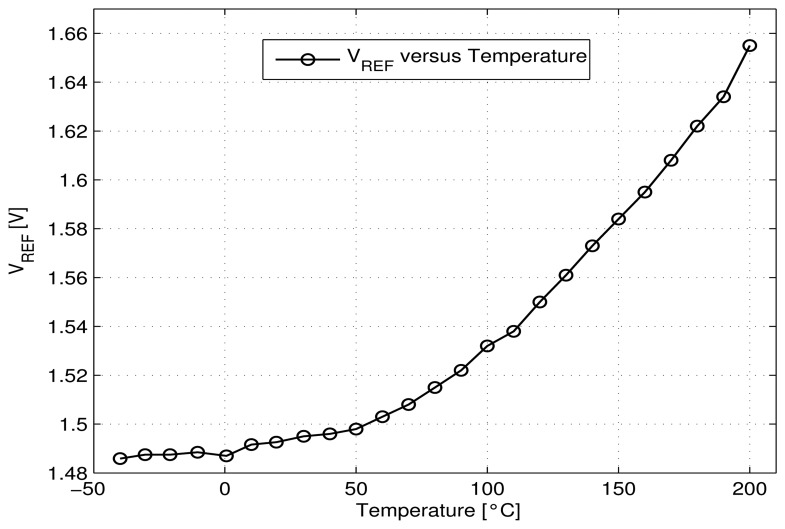
Measured voltage reference *V_REF_versus* temperature.

**Figure 9.**
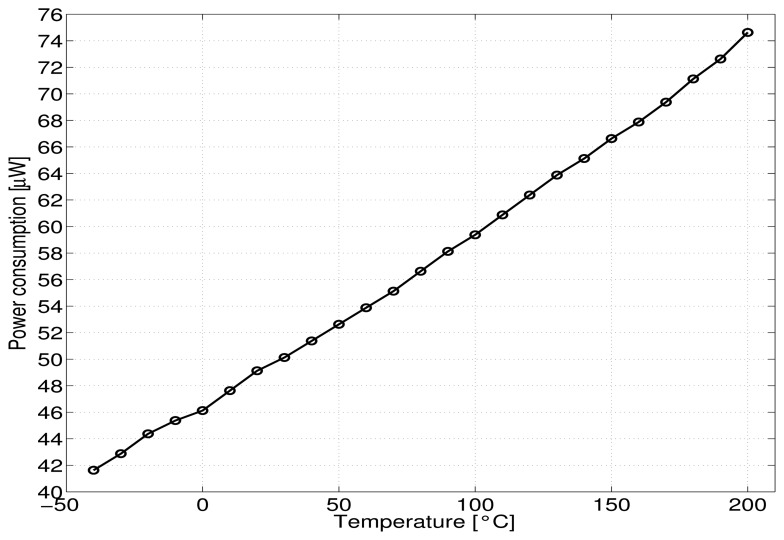
Measured current consumption *versus* temperature.

The experimental voltage reference generates a mean reference voltage of about 1.5 V (Figure 8) with a variation of about 1%, for the temperature range of −40–90 °C with a temperature coefficient less than 133 *ppm*/ °C. This increases to 470 *ppm*/ °C for the large tested temperature range of −40–200 °C with a maximum variation of 10%. The maximum power dissipation is less than 42 *μW* at the lower temperature of −40 °C, about 50 *μW* at room temperature and only 75 *μW* at a high temperature of 200 °C (Figure 9).

The 10% increase of the reference voltage value *V_REF_* textitversus temperature can be explained through the increase of the subthreshold current and parameters *n, U_T_* and (1/*β_Mn_*) shown in the Equation (9). The decrease of the absolute values of threshold voltages of transistors *V_thn_* and |*V_thp_*| with temperature [8] helps (but not sufficiently) to limit the increase of *V_REF_* value versus temperature. To first order, the power dissipation of the circuit is equal to “*V_DD_*.(m+2).*I_B_*” and according to Equation (8) is proportional to the term n.*U_T_* (∝ n.T). This explains the linear variation with temperature of the power dissipation of the reference voltage (Figure 9).

### 4.2. Measurements under Combined High Temperature and Radiation Exposure

**Figure 10.**
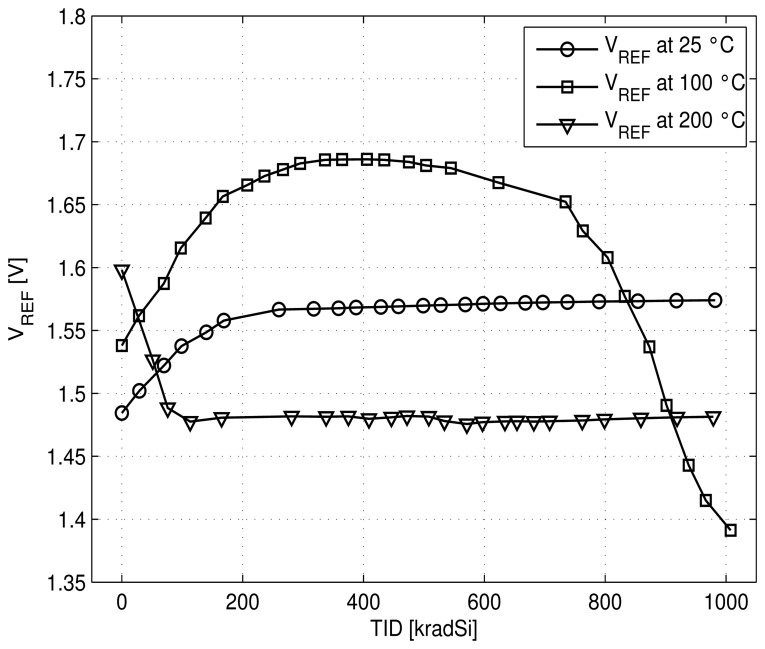
Measured voltage reference *V_REF_vs* TID at different temperatures.

**Figure 11.**
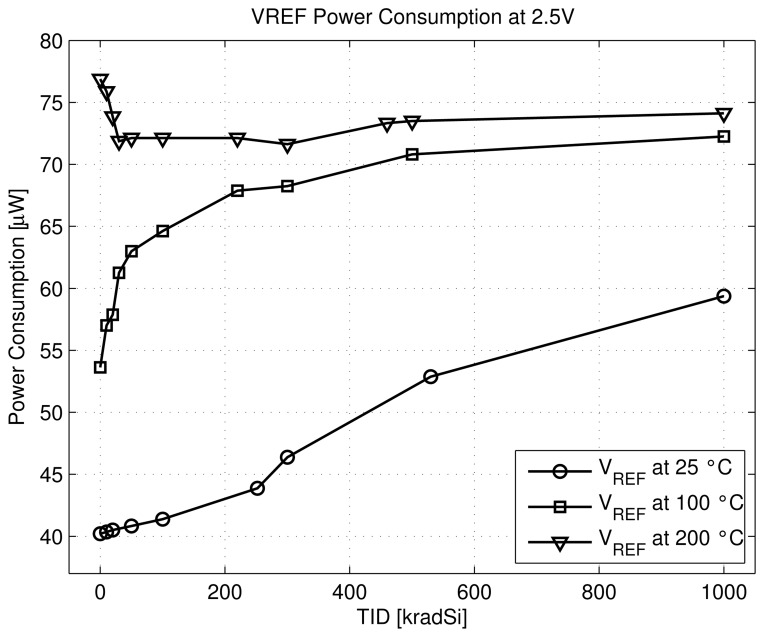
Measured voltage reference power consumption at 2.5 V power supply, in the same conditions as in Figure 10.

Six chips were exposed to gamma-rays radiation during one week with a dose rate of 10 krad/h and regularly measured. Two chips were kept at room temperature, two heated at 100 °C during radiation and the last two chips at 200 °C. The voltage reference value remains about the expected voltage of 1.5 V with a maximum increase of ±5% shift for room and 200 °C temperature. At 100 °C the voltage reference value increases with total dose up to 400 krad (Si) and starts to decrease at higher dose (Figure 10). The power consumption of the voltage reference increases with radiation and temperature, except for the highest heating temperature during radiation for which the power remains stable at about 75 *μ*W upon radiation (Figure 11). Similar trends are observed for all measured chips. Similarly to temperature case, we can express the radiation dependencies of the voltage reference circuit. This is about 25 *ppm*/krad for room temperature up to 1 Mrad (Si).

### 4.3. Discussion of Measurement Results

In order to understand the effect of the combination of radiation and high temperature on the designed voltage reference circuit, the threshold voltages *Vth_n,p_* for the used SOI transistors were extracted in the same conditions (irradiation + temperatures). Gamma-rays radiation is known to result in oxide and interface charges build-up (*N_ox_* and *N_it_*). They shift the threshold voltages as (*Vth_n_* ∝ (*N_it_* – *N_ox_*) and |*Vth_p_*| ∝ (*N_it_* + *N_ox_*)) and degrade mobilities in transistors [11].

**Figure 12.**
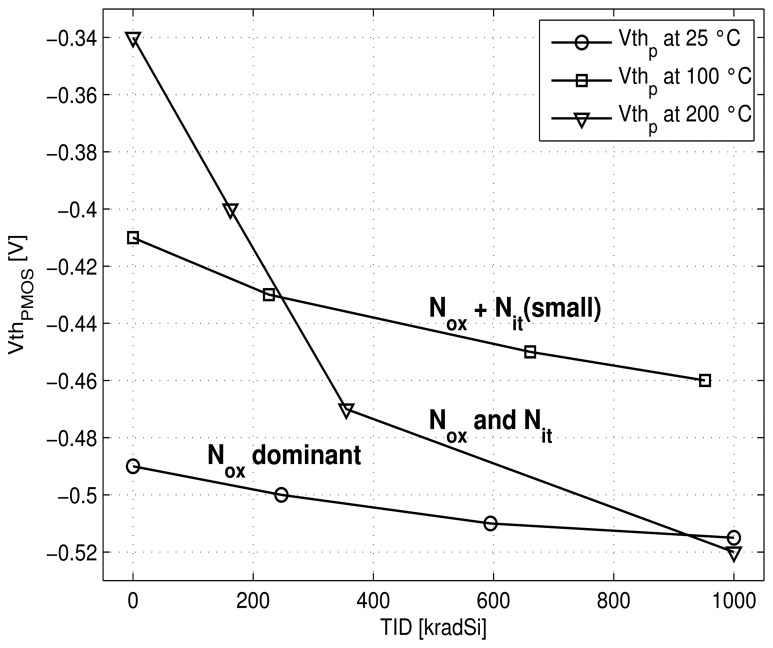
Measured PMOS voltage threshold *vs* TID at different temperatures.

**Figure 13.**
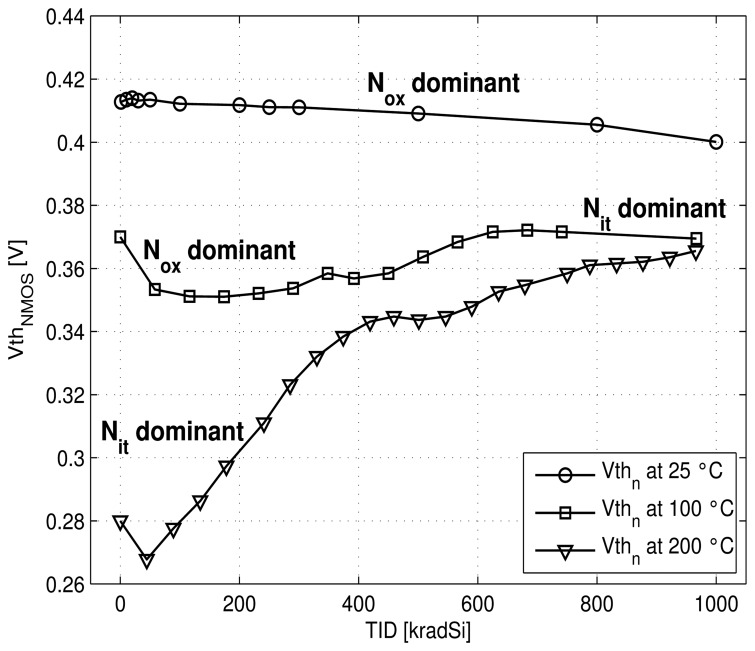
Measured NMOS voltage threshold *vs* TID at different temperatures.

Thus, for PMOS (Figure 12) the absolute |*Vth_p_*| value increases with radiation. This effect is amplified at 200 °C due to higher *N_it_* creation. For NMOS transistor (Figure 13), at room temperature, induced *N_ox_* charges are dominant (versus *N_it_*) and, therefore *Vth_n_* value decreases slightly. At 100 °C a balance between *N_ox_* and *N_it_* occurs and keeps a relatively stable value for *Vth_n_* versus radiation dose. *N_it_* becomes dominant at 200 °C and leads to an increase of the NMOS threshold voltage with dose.

Then, based on the previous expression of *V_REF_* (Equation (9)) and knowing that the parameter n is increased and the carrier mobilities *μ_n_*_(_*_p_*_)_ are degraded both by radiation and temperature [11,14], we can explain the combined effect of radiation and temperature on the circuit (Figures 10 and 11) as follows.

- At room temperature: As the shift of threshold voltages of PMOS and NMOS seems to be small, the increase of *V_REF_* and the power dissipation can be explained by the increase of the parameter n and the decrease of the mobility *μ_n_* (∝ to 1*/β_Mn_*) under radiation.
- At 100 °C: At small dose (less than 400 krad) the same effects as for room temperature occur. For higher dose, the absolute value of the threshold voltage of PMOS increases while the NMOS one remains quasi stable, thus leading to the decrease of the voltage value of *V_REF_* and to stabilize the power dissipation.
- At 200 °C: After an initial decrease of *Vth_n_* and increase of |*Vth_p_*| which is reflected in a decrease of *V_REF_* and the power dissipation, both absolute values of *Vth_n_* and *Vth_p_* increase under radiation. Thus, they compensate each other in the Equation (9) leading to a stable value of *V_REF_* and power consumption.

### 4.4. Comparison with the State of the Art

Table 2 summarizes the performance of the proposed voltage reference circuit and compares it with results of the literature. When compared to our previous work [3], the new circuit is twice less sensitive to temperature variation and the sensitivity to radiation is divided by more than one order of magnitude thanks to: (1) the suitable SOI technology featuring a reduced oxide thickness (5 nm in this work versus 25 nm in the previous work) which limits oxide charge build-up and hence TID degradation; (2) to the new design circuit performed with reasonable margin for the current consumption.

When compared to literature, the proposed circuit operates in a wider range of temperature −40 to 200 °C and radiation (up to 1 Mrad (Si)) than other solutions. Our circuit proposes a high reference voltage value of 1.5 V with a small current consumption (less than 20 *μ*A at room temperature) and achieves a low temperature coefficient for a similar range of temperature.

## 5. Conclusions

In this work we demonstrated an ultra-low power voltage reference circuit, designed in the subthreshold regime of transistors, developed to be used in harsh environment such as biomedical sterilization. This circuit was simulated up to 200 °C using our extended MOS models and was shown to consume about 75 *μ*W only at higher temperature. The design was verified to be robust against radiation effects (using custom model parameters) and the voltage reference value very fairly stable over a large range of process corners and temperature variations. To enhance immunity to total dose effects, the layout has been implemented using specific guidelines and a suitable SOI CMOS technology with thin gate and buried oxides. Measurements have shown a fairly correct operation of such ultra-low power circuit for a large temperature range (from −40 °C up to 200 °C) and under a combination of total ionizing dose (up to 1 Mrad (Si)) and high temperature. The threshold voltages *Vth_n,p_* for the used SOI transistors were extracted under radiation and temperature. They show a significant increase of the absolute value of PMOS threshold voltage with radiation at high temperature, while for NMOS the threshold voltage value *Vth_n_* decreases slightly at room temperature, keeps a relative stable value versus radiation dose at 100 °C and increases at higher temperature 200 °C, depending on the balance between *N_ox_* and *N_it_* build-up. The results fairly support the observed *V_REF_* and power consumption dependences on temperature and total dose radiation.

## Figures and Tables

**Table 1.** Voltage references bias setting and devices dimensions.

**Parameter**	**Description**	**Value**
VDD	Power supply	2.5 V
*I_B_*	Bias current @ 25 °C	2.5*μ*A
(*W/L*)_l_	Size of NMOS *M*_1_	40 *μ*m/0.5 *μ*m
(*W/L*)_2_	Size of NMOS *M*_2_	10 *μ*m/0.5 *μ*m
(*W/L*)_3–6_	Size of PMOS *M*_3_,*M*_4_,*M*_5_ and *M*_6_	10 *μ*m /0.5 *μ*m
(*W/L*)_7–8_	Size of PMOS *M*_7_ and *M*_8_	25 *μ*m/0.5 *μ*m
(*W/L*)*_p_*	Size of PMOS *M_p_*	200 *μ*m/0.5 *μ*m
(*W/L*)*_n_*	Size of NMOS *M_n_*	20 *μ*m/1 *μ*m
(*W/L*)_9_	Size of NMOS *M*_9_	5 *μ*m/0.5 *μ*m
(*W/L*)*_st_*	Size of NMOS *M_st_*	5 *μ*m/1 *μ*m
*R_B_*	*R_B_* resistor value	25 kΩ
*R*_1_	*R*_1_ resistor value	50 MΩ
*R*_2_	*R*_2_ resistor value	20 MΩ
*R_st_*	*R_st_* resistor value	1.5 MΩ

**Table 2.** Performance comparison with similar voltage references described in the literature.

**Parameter**	**This Work**	**Previous work [3]**	**Leung [5]**	**Malcovati [15]**	**Ivanovic [16]**	**Gromov [17]**
Technology	0.13 *μm* SOI	1 *μ*m SOI	0.6 *μ*m Bulk	0.35 *μm* BiCMOS	–	0.13 *μ*m Bulk
Supply voltage	2.5 V	5 V	1.4 to 3 V	1 V	–	1.4 V
Reference voltage (V)	1.5	1.8	0.333	0.54	1.6	0.405
Temperature range (°C)	–40–200 133 [–40, 90 °C]	–40–300 375 [–40, 90 °C]	0–100	0–80	–40–120	0–80
*TC*(*ppm/°C*)	470 [–40, 200 °C]	825 [–40, 200 °C]	94	212	90	200
Consumption @ 25 °C	20 *μ*A	2 *μ*A	9.7*μ*A	92 *μ*A	–	–
*V_REF_* shift due to radiation (*ppm/krad*)	25	426	–	–	140	0.68
Irradiation particles	Gamma-rays	Gamma-rays	–	–	Neutron	X-rays
Maximum *V_REF_* shift due to Combined effect of temperature and radiation up to 1 Mrad (Si)	+10%	–	–	–	–	–
Dose Mrad (Si)	1	1	–	–	1	44
